# Context Matters: Strategies to Improve Maternal and Newborn Health Services in Sub-Saharan Africa

**DOI:** 10.9745/GHSP-D-22-00119

**Published:** 2022-04-28

**Authors:** Florina Serbanescu, Margaret E. Kruk, Sunday Dominico, Kojo Nimako

**Affiliations:** a U.S. Centers for Disease Control and Prevention, Division of Reproductive Health, Atlanta, GA, USA.; b Department of Global Health and Population, Harvard T.H. Chan School of Public Health, Boston, MA, USA.; c Thamini Uhai, Dar es Salaam, Tanzania.; d Maternal, Newborn and Child Health Team (Consultant), Bill & Melinda Gates Foundation, Accra, Ghana.

## Abstract

Identifying context-appropriate features to maximize the effectiveness of maternal and newborn health interventions in sub-Saharan Africa is a prerequisite to successful programs.

## Key Messages

- Although effective evidence-based interventions at birth to save mother's and newborns' lives exist, challenges related to context-specific health system design and service quality hinder progress in reducing maternal and newborn mortality.
- To reduce maternal and newborn mortality, mothers should labor and deliver at a facility that can perform advanced services and provide appropriate care when complications arise.
- When designing and implementing maternal and newborn health programs, the local context should be centered to maximize the impact of these programs.

See related article by Dominico et al., Nimako et al., and Prasad et al.

Sub-Saharan Africa accounts for only 15% of the world's population but bears nearly 70% of the global burden of maternal deaths and about 40% of global newborn deaths.^1^^,^^2^ With the current pace of progress in the region, the United Nations Sustainable Development Goals, which include a provision to reduce global maternal mortality to less than 70 per 100,000 live births and universal access to sexual and reproductive health services by 2030, cannot be achieved without rapidly increasing availability, access, demand for, and utilization of existing health services.^3^

More than half of maternal and newborn deaths result from complications during childbirth and are largely preventable.^4^ Managing existing or emerging complications in health facilities with adequate capabilities and staff to deliver available interventions for mothers and babies has been found to reduce an estimated 71% of newborn deaths, 33% of stillbirths, and 54% of maternal deaths.^4^ By all standards, effective evidence-based interventions at birth exist, but context may change their effectiveness. Knowledge of the clinical requirements for saving mothers' and newborns' lives do not, however, translate into real progress in many places in sub-Saharan Africa due to challenges in health system design and quality that create intrinsic barriers to timely and appropriate care for many mothers and newborns.^5^ Identifying context-specific features that determine the effectiveness of maternal and newborn health (MNH) interventions in low- and middle-income countries is a prerequisite to successful programs in these settings.

Two articles^6^^,^^7^ in *GHSP* present different implementation approaches to facility-based MNH care using currently available evidence-based interventions. Both examine ways through which delivery systems can ensure that all mothers and newborns receive appropriate care, particularly when complications occur. Timeliness and quality of care were a focus in both studies, as maternal and newborn emergencies require a rapid and well-organized response. Additionally, the overview of the successful long-term program to improve maternal health in Tanzania^8^ provides a detailed account of the program's components— in terms of leadership, health system strengthening, resources, and public awareness—the phased approach, and the lessons learned from its implementation.

## TANZANIA CONTEXT

Tanzania is an example of how concerted political focus on the health system can reduce maternal and child mortality.^9^^,^^10^ Tanzania's government doubled its spending on health, introduced sector-wide basket funding and results-based financing, expanded coverage of key maternal and child health interventions through strengthening primary health facilities, and introduced comprehensive emergency obstetric and newborn care services in health centers.^10^ National commitments toward achieving maternal and child mortality reduction aimed for over 80% of births to take place in health facilities.^10^ With the population dispersed across remote areas and physicians in short supply, the country turned to task shifting for surgical care and staff retention, one of the most comprehensive policies of this kind in sub-Saharan Africa.^11^ Tanzania committed to prioritizing maternal and child health at the highest level, as reflected in the Deliver Now for Women and Children campaign,^12^ launched by the president of Tanzania in 2008. The campaign aimed to accelerate the implementation of key strategies set out in the national roadmap and engage civil society groups and health professionals to promote local-level decision makers and boost public demand for quality MNH services.

Tanzania is an example of how concerted political focus on the health system can reduce maternal and child mortality.

Within these premises, the Program to Reduce Maternal Deaths in Tanzania took shape, scaled up, and significantly reduced maternal and perinatal mortality in one of the most resource-poor regions in the country.^6^^,^^8^ While the Program's main focus was on increasing availability of and access to emergency obstetric and newborn care (through improvements in facility infrastructure, staffing, equipment and supplies, training, and supportive supervision), new components were added over time to further improve quality, strengthen referral systems, and increase demand for maternal health and family planning services.^8^ Initially implemented in district hospitals and selected health centers, the Program expanded to support close to half of the dispensaries in the region, in recognition of the fact that they were the most used level of facility for primary preventive services and delivery care. Periodic assessments of the Program's components were conducted to capture the provision, quality and utilization of services, and their coverage and impact.^8^ Results were reviewed with implementers, district, regional, and national stakeholders and were fed back into the Program's design, implementation, and management. More importantly, the Program's longevity ensured sufficient time for planning, implementation, and management to adapt to local context, mature, and yield substantial reductions in maternal and perinatal mortality.

## KENYA CONTEXT

Nimako et al.^7^ illustrate a different approach taken to redesign health service delivery for mothers and newborns in Kenya. In Kakamega County, in western Kenya, there was a strong commitment to significantly reduce maternal and newborn mortality by reorganizing the health system to ensure that the right care is provided by the right provider in the right place at the right time to maximize outcomes and not just access. Specifically, for maternal and newborn health service delivery redesign (MNH SDR), this means that all births would occur in or close to advanced facilities that can provide immediate definitive care for complications, including capacity for cesarean deliveries, blood transfusion, and care for sick mothers and newborns, while the primary care level is strengthened to provide quality antenatal, postnatal, and newborn care.^13^

In Kenya, leadership at the highest political level demonstrated the ambition to drastically reduce maternal and newborn mortality by reorganizing the health system to ensure that the right care is provided by the right provider in the right place at the right time.

Due to the complexity of systems-level reform and the variability in the maturity of health systems in different settings, a one-size-fits-all approach cannot be used in implementing MNH SDR. This is why a feasibility assessment of MNH SDR was necessary in Kakamega County, as the first step toward defining locally specific program needs and adaptations. This assessment involved geographic analyses of distance to hospital, facility and provider surveys to determine gaps in provision of high-quality care, and stakeholder engagement and community focus groups to understand user preferences, learn perceptions about the concept of MNH SDR, and receive input for program design. The feasibility assessment thus helped define the existing system assets that would support the implementation of the MNH SDR policy and the gaps that needed to be closed before the policy was operationalized. The decision to proceed with the program was taken by the government only after careful consideration of the feasibility assessment results. This decision was followed by a government-led codesign process that ensured that a logical rollout plan was devised and locally appropriate strategies were defined to address the human resource, financing, transportation, and infrastructure gaps identified during the feasibility assessment.

The programs implemented in 2 rural settings in Tanzania^6^^,^^8^ and Kenya^7^ share a common vision and drive toward designing well-planned interventions after detailed exploration of health and policy systems at the district level. They both aim to increase availability and quality of timely definitive care for emergencies: emergency obstetric care for mothers and advanced newborn care for sick babies. They demonstrate the need for engaging the highest levels of policy to drive meaningful change and highlight the centrality of local context in the design and implementation of MNH programs. The Tanzania example further exemplifies how adaptive planning over the course of a program is necessary to sustain impact. The Kenya example on the other hand underscores the importance of conducting a feasibility assessment to adequately plan and prepare for implementation and ensure the best program-context fit to maximize outcomes.

## CONCLUSION

Taken together, these analyses demonstrate welcome progress achieved in 2 countries where maternal, newborn, and child health still lags behind national and global commitments. They document locally designed and context-appropriate interventions tailored for maximum impact. They also document the need to set ambitious goals, with commitment from the highest levels of leadership to drive meaningful change. And both demonstrate the urgent need to rethink the models of MNH service provision in sub-Saharan Africa. To save the most lives, countries need to shift from strategies that solely focus on extending access to facility birth to strategies that emphasize access to timely, high-quality advanced care for emergencies to maximize survival. Lessons learned from these experiences can inform policy makers and program managers in other low- and middle-income settings where similar approaches could be used to improve and sustain the utilization and quality delivery of MNH services.

## Notes

**Peer Reviewed**
